# Structural validity of the Dutch version of the disability of arm, shoulder and hand questionnaire (DASH-DLV) in adult patients with hand and wrist injuries

**DOI:** 10.1186/s12891-018-2114-7

**Published:** 2018-06-30

**Authors:** M. E. van Eck, C. M. Lameijer, M. El Moumni

**Affiliations:** University of Groningen, University Medical Center Groningen, Department of Surgery, Groningen, The Netherlands

**Keywords:** Disability arm shoulder hand, Hand, Wrist, Structural validity, Confirmatory factor analysis, Bifactor model

## Abstract

**Background:**

Fractures of the hand and wrist are one of the most common injuries seen in adults. The Disabilities of the Arm, Shoulder and Hand (DASH) questionnaire has been developed as a patient-reported assessment of pain and disability to evaluate the outcome after hand and wrist injuries. Patient reported outcomes (PROs) can be interpreted as pain, function or patient satisfaction. To be able to interpret clinical relevance of a PRO, the structural validity and internal consistency is tested. The Dutch version of the DASH has not yet been validated.

The aim of this study was to evaluate the structural validity and the internal consistency of the existing Dutch version of the DASH. The relevance of reporting subscale scores was investigated.

**Methods:**

This study was a retrospective analysis of cross-sectional data of 370 patients with an isolated hand or wrist injury. Adult patients aged 18 to 65 years treated conservatively or surgically were included. Patients unable to understand or read the Dutch language were excluded. Confirmatory factor analysis was used to investigate the structural validity, while Cronbach’s alpha and coefficient omega were used to assess internal consistency.

**Results:**

All investigated models (a single factor model, a 3-correlated factor, and a bifactor model) were associated with a good model fit. Both the single factor and the 3-correlated factor model were associated with factor loadings of at least 0.70. In addition, the covariance between the factors in the 3-correlated factor model was positive (at least 0.89) and statistically significant (*p* < 0.001). In the bifactor model, the additional value of subscales was limited as the items loaded high on the general factor but low on the subscale factors.

**Conclusion:**

This study indicates that the Dutch version of the DASH should be considered as an unidimensional trait. A single score should be reported.

## Background

Hand and wrist injuries are commonly seen in adults [1–4]. About 20% of all visits to the emergency departments are due to hand and wrist injuries [5, 6]. Considering the ageing of the population, the incidence for these injuries is going to grow [7, 8].

The prevalence of chronic pain following distal radius fractures is reported to be as high as 30%. Of these patients, 11% report moderate to very severe pain 1 year after the initial injury [9, 10]. Longterm disability largely affects elderly patients, of whom 46–95% report some degree of disability 1 year following the initial accident, and 7–16% even report moderate to very severe disability [9, 10]. Aforementioned complaints may result in patients’ inability to perform daily activities.

The International Classification of Functioning, Disability and Health, the ICF, provides a standard language and framework for the description of functioning and disability [11]. In the ICF, functioning problems are classified in three areas: Impairments, Activity limitations and Participation restrictions. The broad concept of disability can refer to any or all areas of functioning in the ICF. Patient reported outcomes (PROs) are one of the most common techniques to assess the different facets of functioning. These outcomes are reported by patients and not defined by an observer [12]. They may be used in clinical decisionmaking, as well as in health care policies and reimbursement decisions [13, 14]. To ensure a PRO can be used in clinical practice for these abovementioned functions, they have to be validated. [14]

Recently, recommendations for a core set of domains for standardized reporting in distal radius fracturs have been published. [15] Pain and function were considered as primary domains.

In every day practice, mostly traditional outcome measures are used to determine results of treatment. For hand and wrist injuries these include physical examining, range of motion, grip strength and radiographic imaging. These examinations mainly reflect aspects of disability in bodily functions. However, the traditional outcome measures are “clinician based” and do not correlate well with aspects that patients find important, such as activity limitations [16]. Therefore, PROs are increasingly used to evaluate the result of treatment and rehabilitation, also in patients with hand and wrist injuries.

The American Academy of Orthopedic Surgeons, the Council of Musculoskeletal Specialty Societies and the Institute for Work and Health developed a questionnaire which reflects the impact of injury on function of a variety of upper extremity musculoskeletal disorders or injuries and developed the Disabilities of the Arm, Shoulder and Hand, questionnaire (the DASH) [17]. The DASH is a 30-item, self-report questionnaire to measure physical function and symptoms in people with musculoskeletal disorders of the upper limb [17]. The questionnaire consists of 3 subscales: a physical subscale, a symptoms subscale and the psychosocial subscale. The DASH has been translated and adapted into several languages [18–32].

In literature exploratory factor analyses (EFA) have been conducted by several authors in different languages to examine the underlying factors of the DASH questionnaire [22, 23, 33]. EFA is a data-driven method without making specifications about the number of and relationships between the latent factors. This approach is used as an exploratory technique. In contrast, confirmatory factor analysis (CFA) requires strong empirical or conceptual grounds to guide the specification and evaluation of the structure of the model in advance [34]. To date, only two studies reported on CFA of the DASH, which were performed on the Italian and American version of the DASH [35, 36].

In this study, the structural validity of the existing translated Dutch version of the DASH (DASH-DLV) was investigated in a patient population with hand and wrist injuries [37]. Particularly, a CFA was conducted, followed by an assessment of internal consistency. Because Veehof et al. already translated the DASH into a Dutch version, we chose not to translate the DASH again [33].

## Methods

### Patients

As described previously, adult patients who sustained an isolated hand or wrist injury in 2012 and 2013 were requested to participate in this cross-sectional study [38]. All patients were treated at a level I traumacenter in the Netherlands, either conservatively or surgically. Included patients had to be 18–65 years of age at the time of injury. Exclusion criteria were unability to speak or read Dutch. All of these patients were invited and sent a paper version of the DASH-DLV, and a reminder after 2 weeks, if needed. The local institutional review board (the Medical Ethics Committee of the University Medical Center Groningen) has reviewed the study protocol and waived further need for approval. In addition, the study was performed in compliance with the principles outlined in the Declaration of Helsinki on ethical principles for medical research involving human subjects [39].

### Disability of arm, shoulder and hand questionnaire

In 1993, the need for a PRO that reflected the impact of a variety of musculoskeletal diseases and injuries of the upper limb on function was independently identified by researchers from the American Academy of Orthopaedic Surgeons’ Outcomes Studies Committee and the Institute for Work & Health [40]. The goal was to develop a self-administered tool that would assess symptoms and physical function at the level of disability, with a focus on physical function, of any or multiple joints or conditions of the upper limb [41]. Item generation and item reduction based on clinimetric and psychometric principles resulted in a 30-item questionnaire [42, 43]. The final 30-item DASH questionnaire includes 21 physical function items, six symptom items and three social/role function items, plus the optional four-item work and sports/performing arts modules.

### Structural validity and internal consistency

**Fig. 1:**
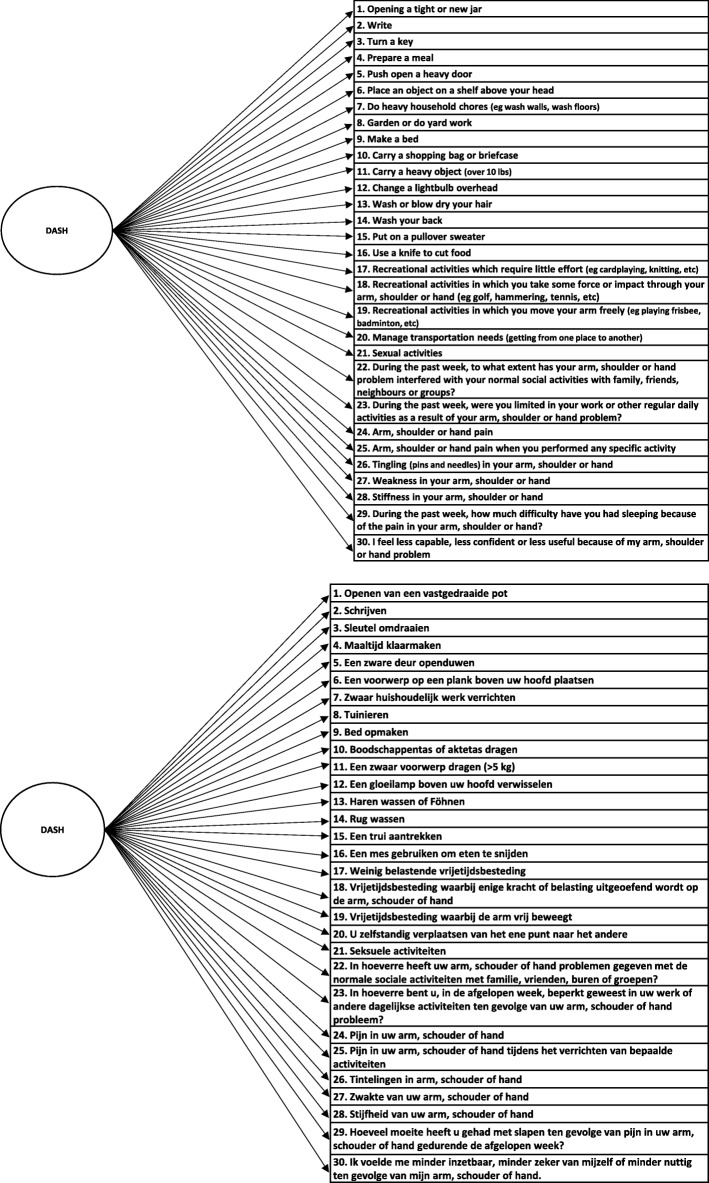
Model 1. a single factor model

**Fig. 2:**
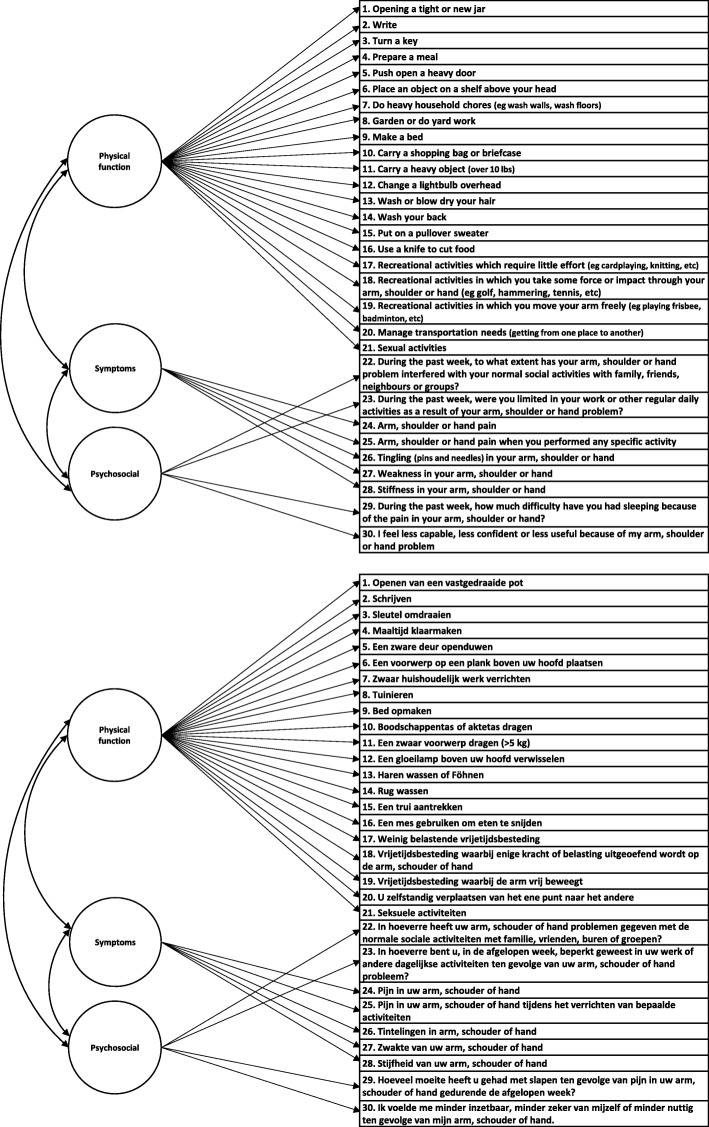
Model 2. a correlated 3-factor model

**Fig. 3:**
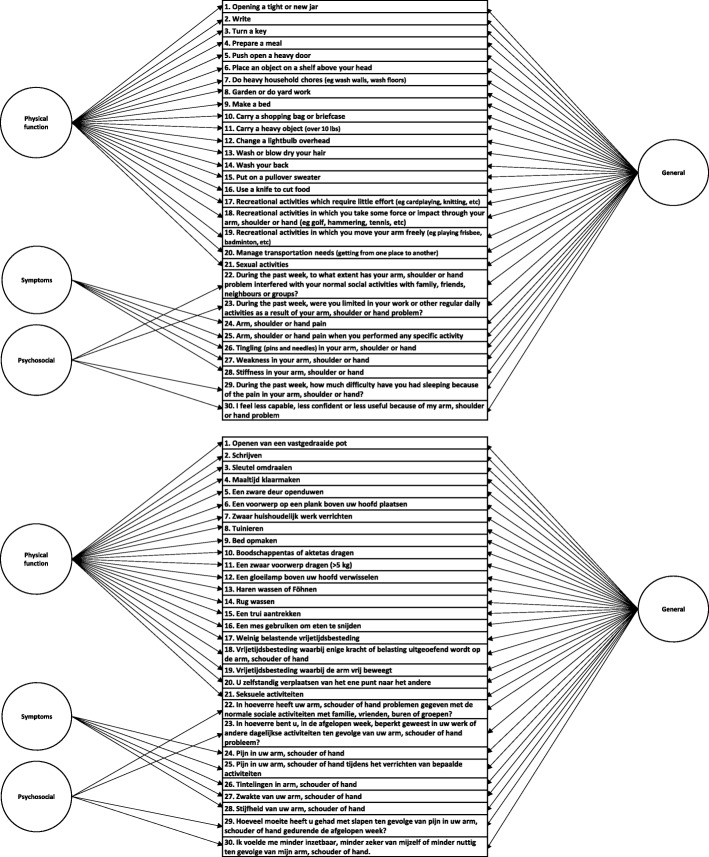
Model 3. a bifactor model

Structural validity, defined as the degree to which scores of an instrument are an adequate reflection of the dimensionality of the construct to be measured, of the DASH-DLV was assessed by CFA [44]. A single factor model of the DASH-DLV (Fig. 1), and a correlated 3-factor model (*Physical Function*, *Symptom and Psychosocial* subscale, Fig. 2) were explored. In addition, a bifactor model was investigated (Fig. 3). A bifactor model includes a general factor associated with all test items and one or more group factors associated with a limited number of items [45] The general factor and group factors are assumed to be uncorrelated. Bifactor models may be used when subscores are expected. Bifactor models are valuable in determining the contribution of subscale scores over and above the general factor [46]. All the investigated DASH-DLV models are presented in Figs. 1, 2 and 3.

Internal consistency examines to what degree the items in a questionnaire are interrelated, and measure the same construct [47]. In this study, the internal consistency was determined after conducting a factor analysis to verify the dimensionality. Two approaches were used. First, Crohnbach’s α was calculated for each (sub) scale. It represents a ratio between the true score variance and the total variance. [47] However, Crohnbach’s α tends to overestimate the reliability of the general factor in a multidimensional data structure and can therefore be misleading in bifactor models [48–50]. Preferably, the coefficient omega total (ω_T_), and omega hierarchical (ω_H_) are used to estimate the internal consistency in a bifactor model [48, 51].

Omega total (ω_T_) is an estimation of the reliability of a factor combining the general factor and the group factor variance. Omega hierarchical (ω_H_) coefficient gives the proportion of variance in scale scores accounted for by a general factor [51]. The coefficient ω_H_ can be extended to estimate the reliability of the group factors, controlling for that part of the reliability due to the general factor in a bifactor model, termed omega subscale (ω_S_) [49, 50]. These coefficients provide useful information to judge whether scores for a group factor can be interpreted with confidence or only the total score (general factor) should be reported. A Cronbach’s α, coefficient omega total, omega hierarchical, or omega subscale of 0.70–0.95 were considered an appropriate reliability.

To evaluate whether our data is ‘unidimensional enough’, two ‘factor strength’ indices were calculated. [50] First, we used coefficient omega hierarchical. [51] A high ω_H_ value indicates that a composite score is reflected by a single common source, i.e. one common factor underlies item responses. In addition, we calculated the Explained Common Variance (ECV), which is the ratio of the variance explained by the general factor, divided by the variance explained by the general factor and the group factors. There are no criteria for ECV to determine whether the data is unidimensional enough, but a higher ECV is seen as a stronger indication for unidimensionality [52].

### Statistical analyses

For validating a questionnaire, there are numerous ways to determine the sample size [53]. In this study, a sample size of 300 cases was chosen, as Comrey and Lee recommend for conducting a factor analysis [54]. Confirmatory factor analyses were conducted using the R’s package *lavaan* [55, 56].

The robust weighed least squares means and variance (WLSMV) estimator with mean- and variance-adjustment was used to fit the models. Completely standardized results were used to report the factor loadings and covariances.

For each model, the χ^2^ goodness-of-fit statistic was computed as the test of global fit. However, this statistic calculation is sensitive to the sample size. Four other commonly used fit indices were calculated as well to evaluate model fit. These indices included the comparative fit index (CFI), the Tucker-Lewis Index (TLI), the root mean square error of approximation (RMSEA), and the standardized root mean square residual (SRMR). A CFI and TLI close to 0.95 or higher, a RMSEA close to or less than 0.06, and a SRMR close to or less than 0.08 were considered as adequate model fit [57].

## Results

As described previously, a cohort of 466 patients who sustained isolated hand or wrist injury were eligible, of which 370 (79.4%) patients (188 males and 182 females) participated in the current study, with a mean age of 43.6 (SD = 14.2) years [38]. The majority of the hand and wrist injuries (82%) were treated conservatively. A large proportion of the injuries were fractures, mainly of distal radius (130/334) (Table 1). The follow-up time ranged from 1 to 25 months. The DASH-DLV questionnaire was completely filled in by 329 of the responders (88.9%). Sixteen patients (4.3%) had a missing response on the item “sexual activities”. The rest of the items were missing in less than 3%. Total scores could be calculated according to the DASH manual for all patients [40].

**Table 1:** Frequencies of hand and wrist injuries

Injury	Frequency (%)
Distal radius fractures	132 (35.7)
Carpal fractures	44 (11.9)
Metacarpal fractures	61 (16.5)
Phalangeal fractures	99 (26.8)
Finger joint dislocations	31 (8.4)
Others	3 (0.8)
Total	370

The 3 CFA models with corresponding fit statistics are presented in Table 2, the standardized factor loadings are presented in Table 3. Although associated with a significant χ^2^ goodness-of-fit (584.83, df = 405, *p* < 0.001) adequate levels of absolute (RMSEA and SRMR) and incremental fit indexes (CFI and TLI) were calculated for Fig. 1. All factor loadings for this model were higher than 0.70 (Table 3).

**Table 2:** Fit statistics for the 3 CFA models

	Chi-squared goodness of fit	df	*p*	RMSEA (90% confidence interval)	SRMR	CFI	TLI
Model 1	584.83	405	0.000	0.035 (0.028–0.041)	0.055	0.993	0.992
Model 2	498.12	402	0.001	0.026 (0.017–0.033)	0.050	0.996	0.996
Model 3	419.96	375	0.054	0.018 (0.000–0.027)	0.041	0.998	0.998

*df* degrees of freedom, *p* = *p* value, *RMSEA* root mean square error of approximation, *CFI* comparative fit index, *TLI* tucker-Lewis index, *SRMR* standardized root mean square residual

**Table 3:** Factorloadings of the 3 different confirmatory factor models

Correlated factor model					Bifactor model			
Model 1		Model 2			Model 3			
1-factor		3-factor			Bifactor (3-factor)			
Item	λ_1_	λ _1_	λ _2_	λ _3_	λ _G_	λ _g1_	λ _g2_	λ _g3_
	DASH	Physical	Symptoms	Psychosocial				
1	0.84	0.85			0.84	0.13^a^		
2	0.78	0.79			0.79	−0.13^a^		
3	0.82	0.83			0.83	−0.04^a^		
4	0.88	0.89			0.88	−0.17^a^		
5	0.85	0.85			0.85	0.24^a^		
6	0.90	0.90			0.90	−0.05^a^		
7	0.91	0.92			0.91	0.12		
8	0.92	0.93			0.93	0.06^a^		
9	0.90	0.90			0.90	−0.07^a^		
10	0.93	0.94			0.89	0.36		
11	0.91	0.92			0.88	0.40		
12	0.88	0.88			0.88	−0.10^a^		
13	0.85	0.85			0.83	−0.34^a^		
14	0.85	0.85			0.85	−0.18^a^		
15	0.89	0.90			0.88	−0.38^a^		
16	0.85	0.85			0.86	−0.17^a^		
17	0.88	0.90			0.89	−0.16^a^		
18	0.89	0.90			0.90	0.03^a^		
19	0.91	0.92			0.92	0.07^a^		
20	0.88	0.89			0.87	−0.25^a^		
21	0.80	0.81			0.80	−0.12^a^		
22	0.86			0.89	0.86			0.08^a^
23	0.90			0.94	0.89			0.37^a^
24	0.90		0.93		0.83		0.57	
25	0.91		0.95		0.86		0.37	
26	0.71		0.75		0.69		0.25	
27	0.89		0.95		0.88		0.22	
28	0.80		0.84		0.78		0.30	
29	0.86			0.89	0.86			0.09^a^
30	0.91			0.94	0.90			0.36^a^
(Σλ^2^)	25.10	16.28	3.94	3.35	22.31	0.88	0.66	0.28
ECV					0.92			
α	0.97	0.96	0.91	0.88				
ω_T_					0.98†	0.97†	0.91†	0.90†
ω_H_					0.96†			
ω_s_						0.01†	0.26†	0.11†

Factor loadings are completely standardized estimates. All factor loadings were statistically significant except those marked with ^a^. *G* general factor, *g* group factor, λ factor loading, *ECV* explained common variance, *α* cronbach’s alpha, *ω*_*T*_ omega total, and *ω*_*H*_ omega hierarchical, *ω*_*S*_ omega subscale. †*p* < 0.001

Figure 2 also yielded a significant χ^2^ goodness-of-fit value (498.12, df = 402, *p* = 0.001), but satisfactory absolute and incremental fit indexes. In Fig. 2, all items loaded high on one of the three correlated subscale factors *Physical, Symptoms* and *Psychosocial*. The factor loadings ranged from 0.75 to 0.95. Only 5 and 4 items loaded on subscale factors *Symptoms* and *Psychosocial*, respectively. The covariance between the correlated factors was positive and statistically significant (*Physical* versus *Symptoms* = 0.89, *Physical* versus *Psychosocial* = 0.94, *Symptoms* versus *Psychosocial* = 0.92, all *p*-values< 0.001).

The bifactor Fig. 3 was associated with good levels of model fit indexes: χ^2^ value of 419.96 (df = 375, *p* = 0.054), RMSEA = 0.018, SRMR = 0.041, CFI = 0.998, TLI = 0.998.

However, in Fig. 3, many items loaded high (ranging from 0.69 to 0.93) on the general factor, but low on the subscale factors (Table 3). As an example, the correlated model (Fig. 2) suggests that item 8 ‘Garden or do yard work’ was a strong indicator of the *Physical* subscale (i.e. a factor loading of 0.93). In contrast, Fig. 3 (the bifactor model) indicated that item 8 was a weak indicator (i.e. a factor loading of 0.06).

The ECV is 0.92 in Fig. 3. The factor strength indexes are also presented in Table 3. The coefficient ω_H_ was high for the general factor (0.96), but ω_S_ was low for the group factors (*Phyical, Symptoms* and *Psychosocial*; which were 0.01, 0.26 and 0.11 respectively). These results indicate that a large portion of the total variance is explained by the general factor, and only a very small portion of the total variance is explained by subscale factors. Regarding internal consistency, Crohnbach’s α of the single and the 3-correlated factor models (Figs. 1 and 2) were high, ranging from 0.88 to 0.97. These findings suggest that the DASH-DLV measures a single factor model and that it is not beneficial to report subscale scores.

## Discussion

The various CFA models were used to clarify how the items of the DASH-DLV relate to each other, and to explore if there were any subscale scores that should be used when scoring the questionnaire. This study suggests that the DASH-DLV reflects a unidimensional trait, and thus reporting subscale scores in the Dutch translation of the DASH is of very limited value and should be avoided.

The Upper Extremity Collaboration Group used principle component analysis to determine the dimensionality of the DASH. Although a two-factor model explained more variance and the scree plot suggested two factors, a one-factor model is recommended given its simplicity [40].

While principal component analysis aims to explain all variance in the data set, making it most appropriately applied as a data reduction technique, EFA is used to only explain the common variance of all items, discovering a set of yet unknown latent variables based on the data. In contrast, confirmatory factor analysis makes it possible to test whether the data fit a prehypothesized factor structure based on empirical data or theory, making this technique more appropriate to confirm the factor structure (i.e. dimensionality) of a questionnaire. The choice for a particular method of factor analysis is crucial, because the different techniques have different assumptions about the data and answer different research questions [58].

In this study, we used CFA since our reseach question was to confirm the factor structure of the DASH-DLV. To our knowledge, only two studies have conducted CFA to examine the DASH questionnaire [35, 36]. Franchignoni et al. investigated the factor structure of the Italian version of the DASH [35]. After an exploratory approach, the 3-factor structure showed adequate fit, nonetheless with some misfitting items. A 1-factor model of the DASH was not confirmed as indicated by poor fit statistics.

In the American version, Lehman et al. also tested a 3-factor model after excluding item 20 and 21 because of their unacceptably low factor loadings [36]. Although the TLI and SRMR values indicated good fit, the CFI and RMSEA do not. In addition, they found high interfactor correlations (> 0.83).

All models in our study yielded adequate fit to the data (Table 2). Both Fig. 1 (one-factor) and Fig. 2 (3-correlated factors) showed high and statistically significant factor loadings. However, the subscales *Symptoms* and *Psychosocial* of Fig. 2 included only 3 and 2 items, respectively, potentially compromising the coverage of the construct’s theoretical domain. All items in the bifactor model (Fig. 3) were associated with high factor loadings on the general factor, but low on the group factors. Bifactor analysis allows researchers to empirically examine the appropriateness of using subscales. To date, research in assessing the structural validity of DASH has not included bifactor models.

Several important findings support that the DASH-DLV is sufficiently unidimensional. First, the covariance between the 3 correlated factors in Fig. 2 were all positive and significant, indicating unidimensionality. Second, the factor loadings of the general factor in the bifactor model (Fig. 3) are very similar to the loadings in the single factor model (Fig. 1). Furthermore, the factor loadings are high and statistically significant on the general factor, but substantially lower on the group factors. This suggests that the subscale factor contribution ‘over and above’ the general factor is very limited. [46] Third, the general factor of Fig. 3 accounted for more than 90% (ECV = 0.92) of the common variance, indicating a high degree of unidimensionally. Finally, although the coefficient omega total values estimated in the bifactor model showed very good reliability for the general and subscale factors, the values of omega hierarchical of the general factor differed significantly from the omega subscale of the subscale factors. Omega hierarchical (ω_H_) coefficient gives the proportion of variance in scale scores accounted for by a general factor, whereas the omega subscale represents the reliability estimate of the subscales, accounting for the effects of the reliability due to the general factor in bifactor models [51, 59]. The coefficient omega hierarchical therefore provides useful information on whether scores for subscale factors can be interpreted with confidence, or that only the general factor score should be used. In this study, ω_S_ was very low for the subscale factors (ranging 0.01–0.26), but ω_H_ was high (0.96) for the general factor. This indicates that the subscale factors account for only 1 to 26%, while the general factor accounts for 96% of the variance. This implies that reporting subscale scores in the DASH-DLV is of extremely limited value.

This study has some limitations. The patients who were included mainly experienced distal radius fractures, and were mostly treated non-surgically. This distribution of patients may limit the generalizability of the results. For this study, we only included trauma cases and no elective cases. This may have caused a selection bias towards elderly females. In addition, an existing Dutch translation of the DASH questionnaire was used without employing a translation and culturally adaptation process. However, this Dutch version is widely used and supported by the Institute for Work & Health [37]. Despite these limitations, the response rate was sufficiently high and an adequate sample size was included. There was only a small number of missing values, from which total scores for all patients could still be calculated according to the DASH manual. [40] Finally, future studies should assess validity in more detail, and other measurement properties of the DASH, such as test-retest reliability and responsiveness, should be evaluated.

## Conclusions

In conclusion, this study suggests that the DASH-DLV reflects a unidimensional trait, and thus reporting subscale scores in the Dutch translation of the DASH is of very limited value and should be avoided. Further studies should assess the validity of the DASH-DLV in more detail, as well as other measurement properties, such as test-retest, reliability, measurement error and responsiveness, to ensure reliable interpretation of this patient reported outcome measure in clinical practice.

## Abbreviations

CFA: Confirmatory factor analysis
CFI: Comparative fit index
DASH: Disability of arm shoulder and hand questionnaire
DASH-DLV: Disability of arm shoulder and hand questionnaire Dutch language version
df: Degrees of freedom
ECV: Explained common variance
EFA: Exploratory factor analysis
ICF: The International Classification of Functioning, Disability and Health
PRO: Patient rated outcome
RMSEA: Root mean square error of approximation
SD: Standard deviation
SRMR: Standardized root mean square residual
TLI: Tucker Lewis index
WLSMV: Weighed least squares means and variance
